# Evaluation of the relationship between cytochrome P450 (CYP) 1A2 gene copy number variation and CYP1A2 protein content and enzyme activity in canine liver

**DOI:** 10.3389/fvets.2025.1511341

**Published:** 2025-07-22

**Authors:** Francisco Gonzales Zamora, Hannah Bigelow, Hong Yang, Tania Perez Jimenez

**Affiliations:** ^1^Animal Clinic Veterinary Hospital, Lima, Peru; ^2^Relief Veterinarian, Sahuarita, AZ, United States; ^3^Program in Individualized Medicine, Pharmacogenomics Laboratory, Department of Veterinary Clinical Sciences, College of Veterinary Medicine, Washington State University, Pullman, WA, United States

**Keywords:** veterinary pharmacogenetics, CYP1A2, protein content, gene copy number variation, dog liver bank

## Abstract

Cytochrome P450 (CYP) 1A2 plays a key role in the metabolism of various drugs in dogs. However, the impact of genetic variation on differences in CYP1A2 metabolism among dogs remains unclear. Recent studies have identified variability in the copy number of the CYP1A2 gene, ranging from two to five copies. Additionally, a genetic polymorphism (stop codon) has been identified which results in the expression of an inactive protein, this has been investigated and changes in the pharmacokinetics of some clinically used drugs have been described. If these additional copies are functional, dogs with more CYP1A2 gene copies may exhibit faster drug clearance, potentially affecting appropriate drug dosing. To investigate this possibility, a well-characterized dog liver bank (*N* = 58) was analyzed to determine whether CYP1A2 copy number variation (CNV) correlates with CYP1A2 protein levels and enzyme activity. Real-time PCR was used to assess CYP1A2 CNV, while label-free mass spectrometry measured CYP1A2 protein concentration in liver microsomes. Theobromine N-3 demethylation was examined as a marker of canine CYP1A2 activity using commercially available recombinant CYPs and liver microsomes from dogs treated with isoform-selective enzyme inducers. Only CYP1A1 and CYP1A2 demonstrated the ability to catalyze theobromine N-3 demethylation, and this activity was induced exclusively by β-naphthoflavone. Liver microsome theobromine N-3 demethylation activity showed a moderate correlation with CYP1A2 protein levels (*R*_s_ = 0.46; *p* = 0.0003). Among the 58 liver samples genotyped for CYP1A2 CNV, nine dogs had two copies, 20 had three copies, 23 had four copies, and six had five copies. However, CYP1A2 CNV did not significantly correlate with CYP1A2 protein concentration (*R*_s_ = −0.14; *p* = 0.30) and showed a weak negative correlation with theobromine N-3 demethylation activity (*R*_s_ = −0.45; *p* = 0.00035). These findings suggest that CYP1A2 CNV is not a strong predictor of increased CYP1A2 protein expression or activity. According to the literature, CNV might not be relevant, but the genetic polymorphism (stop codon) could potentially be. The studies available show relationships between the stop codon and protein inactivity in the metabolizing of clinically used drugs. Further studies are necessary to validate these preliminary results.

## 1 Introduction

Cytochrome P450 (CYP450) enzymes play a crucial role in the metabolism of both endogenous compounds and xenobiotics (1). These enzymes facilitate Phase I reactions, which transform these substances into more reactive forms, preparing them for further metabolism and elimination. However, when CYP enzyme activity is diminished or absent, drug clearance can be significantly impaired, leading to increased plasma drug concentrations and a heightened risk of adverse effects and toxicity (2). Several factors influence CYP enzyme activity, including drug interactions, enzyme inhibition, induction, and genetic polymorphisms (3).

Genetic polymorphisms in CYP enzymes contribute to individual variations in drug metabolism. A notable example in dogs is the c.1117C>T (373X) variant in the CYP1A2 gene, which results in a premature stop codon (4, 5). This single nucleotide polymorphism (SNP) was identified during preclinical drug testing in beagle dogs and was associated with significantly increased serum drug concentrations (4, 6). When homozygous (p.373X/X), this mutation (CYP1A2stop) leads to a complete loss of hepatic CYP1A2 protein and its enzymatic function (4, 5). While such coding variants reducing CYP1A2 activity are rare in humans (7), this polymorphism is highly prevalent in certain dog breeds, particularly beagles and Irish wolfhounds (8–10). As a result, affected dogs may experience heightened drug exposure and potential toxicity from medications metabolized by CYP1A2, especially in breeds where the variant is common.

Although canine CYP1A2 is known to metabolize drugs such as caffeine, theophylline, and phenacetin (2), more recent studies have shown that it also contributes to the metabolism of drugs like omeprazole (11). Additionally, CYP1A2 activity can be induced by environmental factors, such as nicotine exposure from secondhand smoke, potentially altering drug metabolism in companion animals (12). While the complete list of drugs metabolized by canine CYP1A2 remains unknown, extensive research has explored its role in processing anti-cancer (13, 14) and antidepressant medications (15).

In humans, CYP1A2 is involved in the metabolism of numerous clinically important drugs, including acetaminophen, phenacetin, lidocaine, clozapine, olanzapine, duloxetine, propranolol, triamterene, tacrine, tizanidine, and zolpidem (16). Caffeine is widely used as an *in vivo* probe to assess CYP1A2 activity, with other probes such as theophylline, duloxetine, tizanidine, and melatonin being used occasionally (16). For *in vitro* studies, henacetin is commonly used, along with ethoxyresorufin and tacrine (17). In dogs, tizanidine has been identified as a selective CYP1A2 probe *in vivo* (18), while phenacetin has been used as both an *in vivo* and *in vitro* probe, particularly in CYP1A2-deficient beagle dogs (19, 20). However, phenacetin’s selectivity for CYP1A2 is moderate, likely due to the involvement of other CYP enzymes, such as CYP2A13, in its metabolism in dogs (21).

Another potential CYP1A2 probe is theobromine, a methylxanthine structurally similar to caffeine and theophylline. In humans, CYP1A2 extensively metabolizes theobromine into 7-methylxanthine, though additional CYP isoforms also contribute, reducing its selectivity (22). To date, the role of canine CYP1A2 in theobromine metabolism has not been investigated.

A recent genomic study revealed that CYP1A2 gene duplication occurs in dogs, with some individuals possessing up to five gene copies (23). However, the functional significance of these additional copies remains unclear. If they lead to increased CYP1A2 expression and enzymatic activity, they could contribute to faster drug metabolism and reduced drug efficacy, potentially resulting in drug resistance. Copy number variation (CNV) in multiple genes is an area of active research in both human and veterinary medicine, as it may contribute to pharmacogenetic variability, phenotypic diversity, and disease susceptibility (24).

The study by Wang et al. (23) conducted a preliminary assessment of CYP1A2 duplication effects on gene expression (mRNA levels) in dog liver samples. The results indicated no significant differences in mRNA levels between dogs with three copies and those with more than three copies. However, the study had a small sample size, and CYP1A2 protein levels and enzymatic activity were not evaluated, leaving questions about functional impact unanswered.

One of the most significant aspects of CYP1A2 research in dogs is its role in comparative pharmacology. Since beagles are widely used as models for human drug metabolism, studies on CYP1A2 in dogs provide valuable insights into enzyme function, hormonal metabolism, and environmental toxin processing (e.g., cigarette smoke exposure). Understanding these factors contributes to safer and more effective drug therapies in both veterinary and human medicine.

This study aimed to investigate how CYP1A2 gene duplication affects CYP1A2 protein levels and enzyme activity using a well-characterized canine liver bank (*N* = 58 dogs). The research specifically hypothesized that higher CYP1A2 gene copy numbers would correlate with increased enzyme expression and activity. Additionally, the study evaluated the potential of theobromine N-3 demethylation to 7-methylxanthine as a CYP1A2 probe reaction in dogs, using recombinant canine CYP enzymes and liver microsomes from dogs treated with CYP inducers.

## 2 Materials and methods

### 2.1 Reagents

Theobromine, 7-methylxanthine, 1,3-dimethyluric acid, NADP^+^, isocitrate dehydrogenase, DL-isocitrate, and magnesium chloride were from Sigma-Aldrich (St. Louis, MO). Recombinant canine CYP bactosomes (CYP1A1, CYP1A2, CYP2B11, CYP2C21, CYP2C41, CYP2D15, CYP3A12, and CYP3A26) were from Xenotech LLC (Lenexa, KS).

### 2.2 Liver microsomes

Liver microsomes were prepared by differential centrifugation according to a previously described method (25) from a bank of dog livers maintained at Washington State University and stored at −80°C. The livers were from 58 healthy adult dogs, including research Beagles (*n* = 25), research hounds (*n* = 12), mixed-breed dogs (*n* = 12), Greyhounds (*n* = 5), and Chihuahuas (*n* = 4). Details for individual animals are provided in Supplementary Table 1. The collection and use of these dog livers was determined to be exempt from review by the Institutional Animal Care and Use Committee at Washington State University since they were purchased from commercial sources, or were tissue collected after euthanasia for reasons unrelated to this research and would otherwise be discarded. The concentration of microsomal protein in each preparation was determined using the bicinchoninic acid assay (Thermo Fisher Scientific, Waltham, MA).

### 2.3 CYP1A2 CNV assay

CYP1A2 CNV genotype was determined by adapting the method reported by Wang et al. (23). Briefly, genomic DNA was extracted from 58 of the 59 dog liver samples (DL-11 had insufficient tissue available). Real-time PCR was performed using the CFX96 Duet Real-Time System with CFX Maestro software (Bio-Rad Laboratories) with custom designed TaqMan gene expression assays (Applied Biosystems). CYP1A2 gene primer sequences were CYP1A2-F-4 5′-GATGTTCCTCCCTAGCGACA-3′ and CYP1A2-R-4 5′-CCATGTTCTCTTGGCTCCAT-3′. The reporter sequence was 5′-VIC-TCCCTGAAGACCTGCCCTGGGT-MGBNFQ-3′ (23). As previously reported by Martinez et al. (26), canine UGT1A was quantified as the reference gene using the predesigned TaqMan gene expression assay Cf03987062_sH (Applied Biosystems) that targeted the highly conserved exon 5 region of the UGT1A gene. Standard curves were generated using synthetic partial fragments (Gene Blocks, IDT) of the CYP1A2 (342 bp) and UGT1A (287 bp) genes that included the real-time PCR binding sites (reference gene sequence—Supplementary Figure 1). Real-time PCR reactions were set up containing 10,000 copies of the reference gene and 500–25,000 copies of the CYP1A2 standard, resulting in molar ratios of 0.5, 1, 1.5, 2, and 2.5 (reference vs. CYP1A2). These ratios corresponded to CYP1A2 gene copy numbers of 1, 2, 3, 4 and 5, as the reference gene invariably contains two copies in the genome (Table 1).

**Table 1:** Copy number ratio relationship to gene copies.

Gene copies	Copy number ratio	Ref (UGT1A) copy number	CYP1A2 copy number
1	0.5	10,000	5,000
2	1	10,000	10,000
3	1.5	10,000	15,000
4	2	10,000	20,000
5	2.5	10,000	25,000

The ratio was obtained by using the amount of copy number generated for CYP1A2 divided by the copy number of the reference gene (UGT1A). The gene copy number was obtained using the reference gene known copy number (2 copies = 1).

Paired (CYP1A2 and reference gene) assays were performed with TaqMan Gene Expression Master Mix (Applied Biosystems) using 4 ng of DNA in each 20 μL reaction volume. Cycling conditions were as follows: 95°C for 10 min, followed by 70 cycles of 95°C for 15 s and 60°C for 1.5 min. The relative CYP1A2 copy number of each standard and genomic DNA samples were expressed as the ∆Ct (Ct of reference gene-Ct of CYP1A2 gene). Results represent the mean and standard deviation of three independent experiments conducted in duplicate.

### 2.4 Liver CYP1A2 protein content and CYP1A2 premature stop codon genotype

Individual liver microsomal CYP1A2 protein concentrations and genotypes for the CYP1A2 premature stop codon mutation (CYP1A2 c.1117C.T; p.Arg373Stop) used here were determined previously for the entire liver bank and reported by Martinez et al. (26). Briefly, CYP1A2 protein concentration was determined by LC-MS/MS proteomic analysis and expressed as pmoles of CYP1A2 protein per mg of microsomal protein. Genotyping was performed with DNA extracted from each liver using a TaqMan allele discrimination assay validated for Sanger sequencing. Of the 58 livers in the bank with available DNA, 46 livers were homozygous reference (p.373 R/R), 11 were heterozygous (p.373 R/X), and one was homozygous variant (p.373 X/X). Notably this latter liver sample (DL-55) also lacked detectable CYP1A2 protein content. CYP1A2 protein content and genotype data are provided for individual samples in Supplementary Table 1. All the experiments were conducted in triplicate on different days and results were averaged.

### 2.5 Theobromine N3-demethylation assay

An assay to measure rates of formation of 7-methylxanthine by N3-demethylation from theobromine by dog liver microsomes and recombinant P450 enzymes was developed based on a previously published method with some modifications (22). The Recombinant canine CYPs were expressed in Bactosomes (1A1, 1A2, 2B11, 2C21, 2C41, 2D15, 3A12, and 3A26; each co-expressed with canine P450 oxidoreductase) treated with corn oil, rifampin, β-naphthoflavone, saline, phenobarbital and clofibric acid and were purchased from Xenotech LLC (Lenexa, KS).

Briefly, standard 100 μL incubations contained enzyme (50 μg liver microsomes or 5 pmole recombinant P450), theobromine (5 mM), and NADPH-regenerating system (0.5 mM NADP^+^, 4 mM D, L-isocitrate, 1 unit D, L-isocitrate dehydrogenase, 5 mM magnesium chloride) in potassium phosphate buffer (50 mM pH 7.4). Reactions were started by adding the NADPH-regenerating system and incubated in a water bath at 37°C for 120 min. Preliminary experiments were conducted to ensure that metabolite formation was proportional to the enzyme concentration and incubation time under these assay conditions. The reaction was stopped by adding 100 μL of ice-cold internal standard (1,3 dimethyl uric acid in 0.5 M perchloric acid), vortexed, centrifuged at 15,000 RCF for 10 min, and the supernatant was then analyzed by HPLC with UV detection at 272 nm.

The HPLC apparatus consisted of an Agilent 1,100 system (Agilent Technologies, Santa Clara, CA). An isocratic mobile phase of 3% acetonitrile with 97% 20 mM potassium phosphate in water (pH 4.5) was pumped at 0.9 mL per minute through a Synergi Hydro-RP 250 × 4.6 mm column (Phenomenex, Torrance, CA) followed by a UV absorbance detector set at 272 nm. Approximate retention times for 7-MX, 1,3 dimethyluric acid (internal standard), and theobromine were 15.2, 23.5 and 24.3 min, respectively. The amount of metabolite formed per minute per mg of liver microsome (or per nmole of CYP) was calculated using a standard curve generated using samples with known concentrations of 7-MX and internal standard dissolved in a blank matrix. All assays were conducted four separate times, and the results averaged.

### 2.6 Statistical analyses

Relationships between CYP1A2 protein concentration, theobromine N3-demethylation and relative CYP1A2 copy number (∆Ct) were evaluated by calculation of the Spearman correlation coefficient to measure the strength of the relationship between the variables giving the data is not normally distributed. To determine the relationship between the CYP1A2 protein content and the estimated copy number, ANOVA on rank transformed data was performed, due to the ranking of the CNV (0–5) related to the protein content. Statistical analyses were performed using Sigma Plot 14.5 software (Systat Software, San Jose, CA). For most statistical tests, a *p* < 0.05 was considered statistically significant. For correlation analyses, an *R*_s_ > 0.70 was considered indicative of a significant relationship between the variables being evaluated.

## 3 Results

### 3.1 CYP1A2 CNV distribution in dog liver bank samples

**Figure 1:**
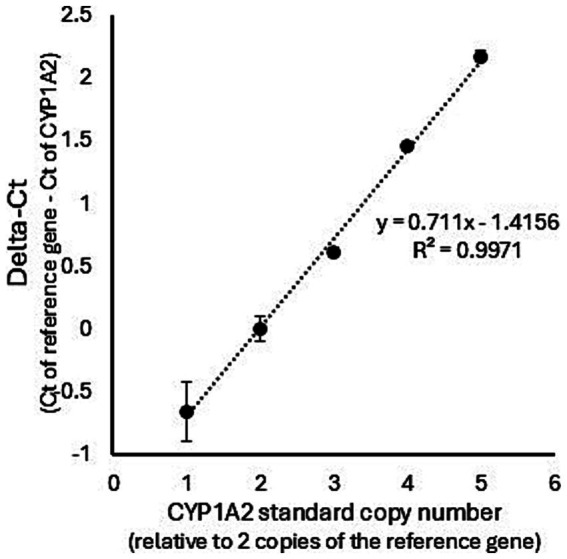
Standard curve showing the relationship between CYP1A2 ∆Ct values and the CYP1A2 standard copy number. The standard curve was generated by the means of triplicated real-time PCR experiments using a synthetic portion of the CYP1A2 gene and a synthetic portion of the highly conserved exon 5 of the UGT1A gene as a copy number control. The relationship between the synthetic standard CYP1A2 copy number and CYP1A2 ∆Ct values was linear (*R*^2^ = 0.997).

**Figure 2:**
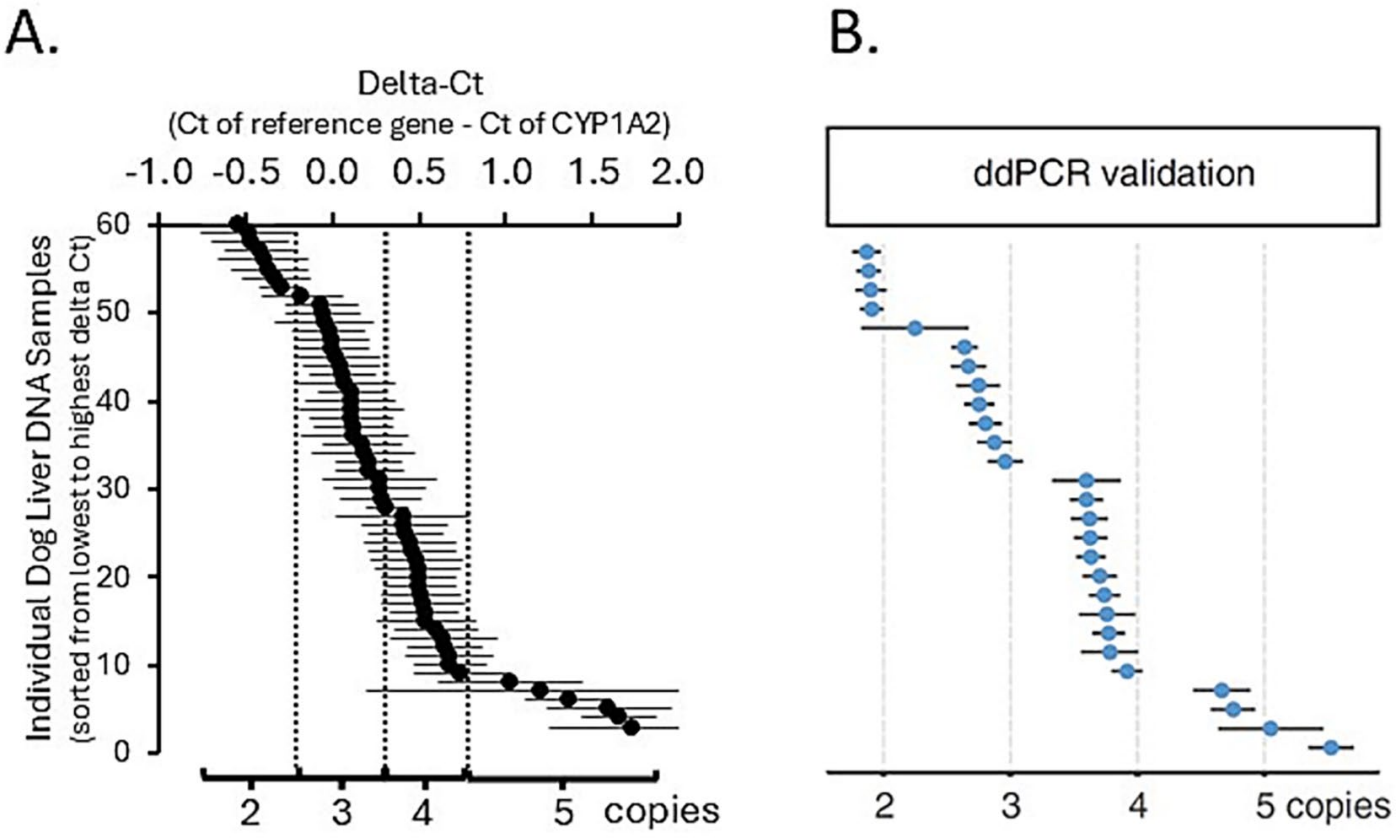
Liver CYP1A2 protein content plotted against CYP1A2 ∆Ct and estimated CYP1A2 copy number **(A)** compared to the figure obtained from Wang et al. (23) **(B)**. This distribution of ∆Ct values and breakpoints was essentially identical.

The CNV distribution in dog livers was investigated. As shown in Figure 1, the relationship between the synthetic standard CYP1A2 copy number and real-time PCR ∆Ct values was linear (*R*^2^ = 0.997) over the assayed range (from 1 to 5 CYP1A2 gene copy equivalents). Assay of dog liver gDNA samples demonstrated a large range of ∆Ct values (−0.053 to 1.72). A plot of these ∆Ct values ordered from lowest to highest (Figure 2A) showed distinct breakpoints that delineated CYP1A2 gene copy number differences (2, 3, 4, and 5 copies) between samples. This distribution of ∆Ct values and breakpoints was essentially identical to that reported previously by Wang et al. (23) (Figure 2B). For our data, ∆Ct values for two copies, ranged from −0.54 to −0.06; for three copies −0.053 to 0.22; for four copies 0.27 to 0.74; and for five copies 1.02 to 1.72. CYP1A2 ∆Ct values and CNV estimates for individual dog samples, as well as demographic information (dog breed and sex) are provided in Supplementary Table 1. Out of 58 dog livers genotyped, nine dogs had two CYP1A2 copies, 20 dogs had three copies, 23 dogs had four copies and six dogs had five copies.

### 3.2 Association of CYP1A2 CNV with liver CYP1A2 protein content and activity

**Figure 3:**
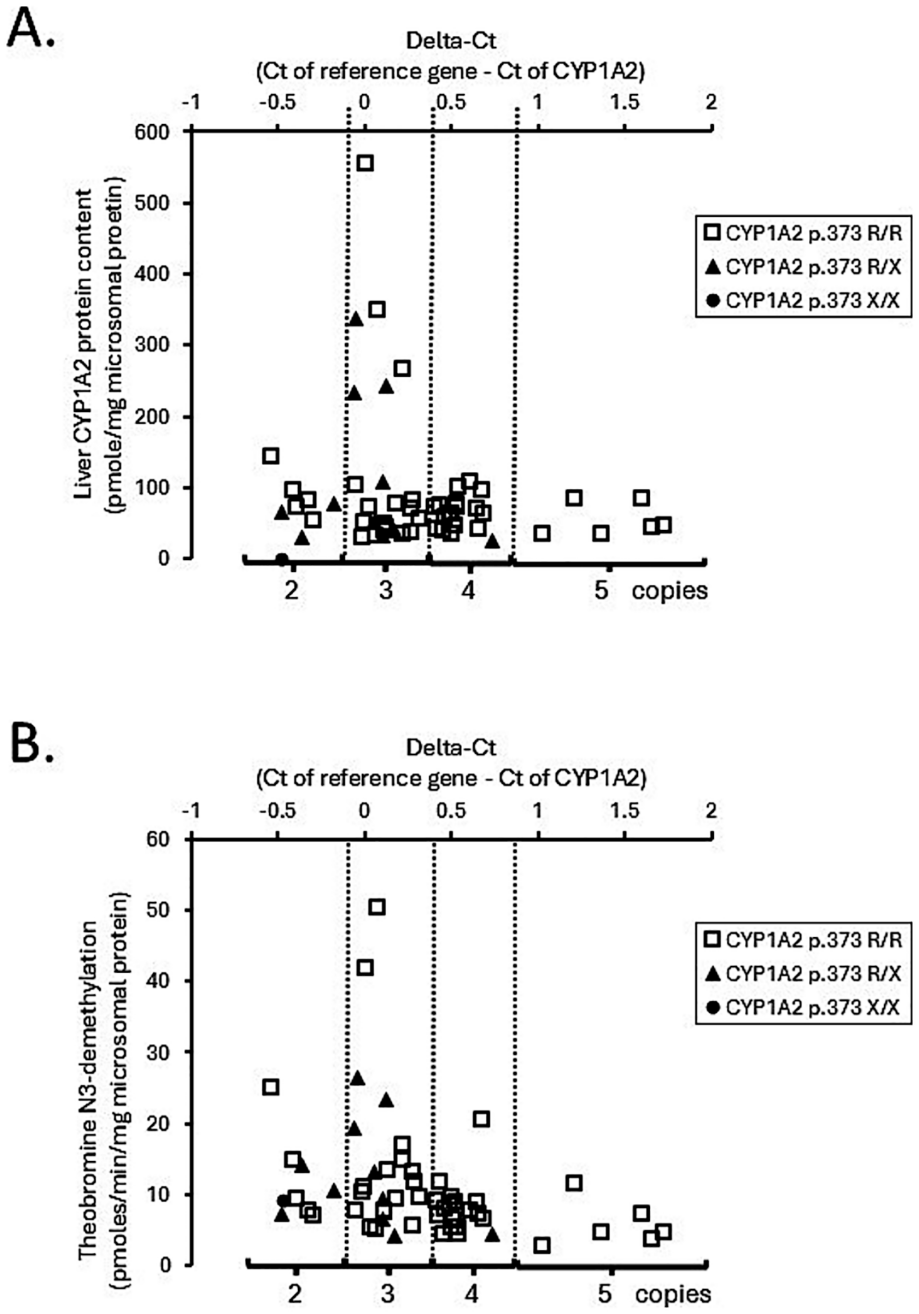
∆Ct of each dog liver sample with estimated CYP1A2 copy number and the liver protein content (pmole/mg microsomes) **(A)** and estimated copy number and Theobromine N3-demethylation (pmoles/min/mg microsomal protein) **(B)** measured by LC-MS/MS in a bank of dog liver microsomes (*N* = 58). Also shown are the premature stop codon (CYP1A2 p.373 R/X) genotypes of each liver sample where it was found that 46 were classified as homozygotes (R/R), 11 were heterozygotes (R/X) and one (LV55) was a mutant (X/X). LV55 showed no detectable CYP1A2 protein content.

The association of CYP1A2 CNV and liver CYP1A2 protein content were assayed and analyzed and Figure 3 shows the association between CYP1A2 ∆Ct values and liver CYP1A2 protein content (Figure 3A) and between CYP1A2 ∆Ct values with theobromine N3-demethylation (Figure 3B) for all liver bank samples (*N* = 58). CYP1A2 ∆Ct values were not correlated with CYP1A2 protein content (Spearman correlation coefficient *R*_s_ = −0.14; *p* = 0.30) and were weakly negatively correlated with theobromine N3-demethylation activities (*R*_s_ = −0.45; *p* = 0.00035). As shown in Table 2 there were also no differences in liver CYP1A2 protein content between livers when grouped by CYP1A2 gene copy number (2, 3, 4, or 5) (ANOVA on ranks; *p* > 0.05). However, there were slightly lower theobromine N3-demethylation activities (Dunn’s test; *p* < 0.05) in livers with five gene copies when compared with those with three gene copies (Table 3).

**Table 2:** CYP1A2 microsomal protein content measured in dog liver bank samples (*N* = 58) with 2, 3, 4, or 5 CYP1A2 gene copies.

Estimated CYP1A2 gene copy number	Microsomal CYP1A2 protein content (pmole/mg microsomal protein)				
	*N*	Median	25%	75%	*p*
2	9	73	42	90	0.74
3	20	63	39	240	
4	23	64	43	76	
5	6	46	37	85	

Shown are the median, 25th to 75th percentile microsomal CYP1A2 protein content values and the *p*-value for comparison between copy number groups by ANOVA on ranks. There is not a statistically significant difference between copy number groups (*p* > 0.05).

**Table 3:** Microsomal theobromine N3-demethylation activities measured in dog liver bank samples (*N* = 58) with 2, 3, 4, or 5 CYP1A2 gene copies.

Estimated CYP1A2 gene copy number	Theobromine N3-demethylation activity (pmole/min/mg microsomal protein)				
	*N*	Median	25%	75%	*p*
2	9	9.5	7.5	14.6	0.009
3	20	^*^10.9	7.7	18.8	
4	23	7.8	5.6	9.7	
5	6	^*^4.8	3.7	8.4	

Shown are the median, 25th to 75th percentile theobromine N3-demethylation activity values and the *p*-value for comparison between copy number groups by ANOVA on Ranks. There is a statistically significant difference between copy number groups (*p* < 0.05). Pairwise group comparisons using Dunn’s test indicated significantly lower (^*^p < 0.05) activities in livers with five gene copies versus three copies.

As shown in Figures 3A,B some of the liver samples carried a genetic variant (CYP1A2 p.373 R/X—11 samples; CYP1A2 p.373 R/X—1 sample) that would reduce CYP1A2 protein content and activity. Consequently, the association analysis was repeated after excluding those 12 samples (46 samples remaining). The results given in Supplementary Tables 2, 3 indicated that neither CYP1A2 protein content or theobromine N3-demethylation activity were associated with CYP1A2 gene copy number. Similar results (no association) were also obtained when this analysis was further restricted to liver samples from beagle dogs (Supplementary Tables 4, 5).

### 3.3 Theobromine N3-demethylation by CYP1A2

**Figure 4:**
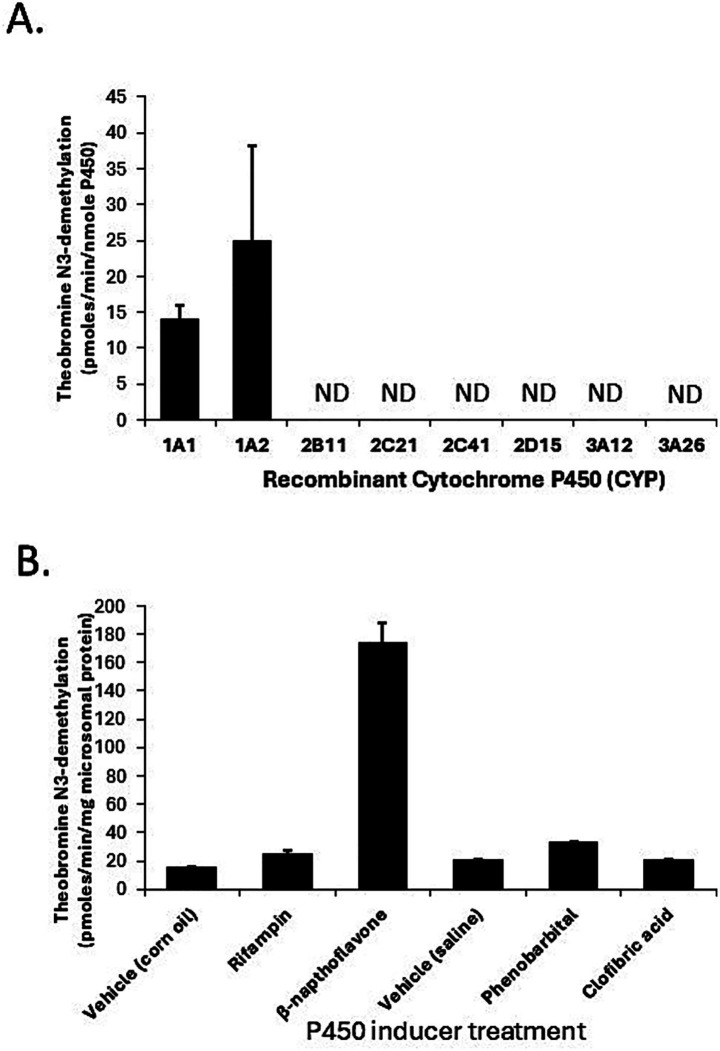
Evaluation of theobromine N3-demethylation as a selective probe for CYP1A2 function in canine liver using all commercially available recombinant canine CYPs **(A)** and liver microsomes from dogs treated with CYP selective inducers (corn oil, rifampin, β-napthoflavone, saline, phenobarbital, and clofibric acid) **(B)**. Only CYP1A1 and CYP1A2 were capable of theobromine N3-demethylation **(A)** while liver microsomal theobromine N3-demethylation activities were only induced in β-napthoflavone treated dogs.

**Figure 5:**
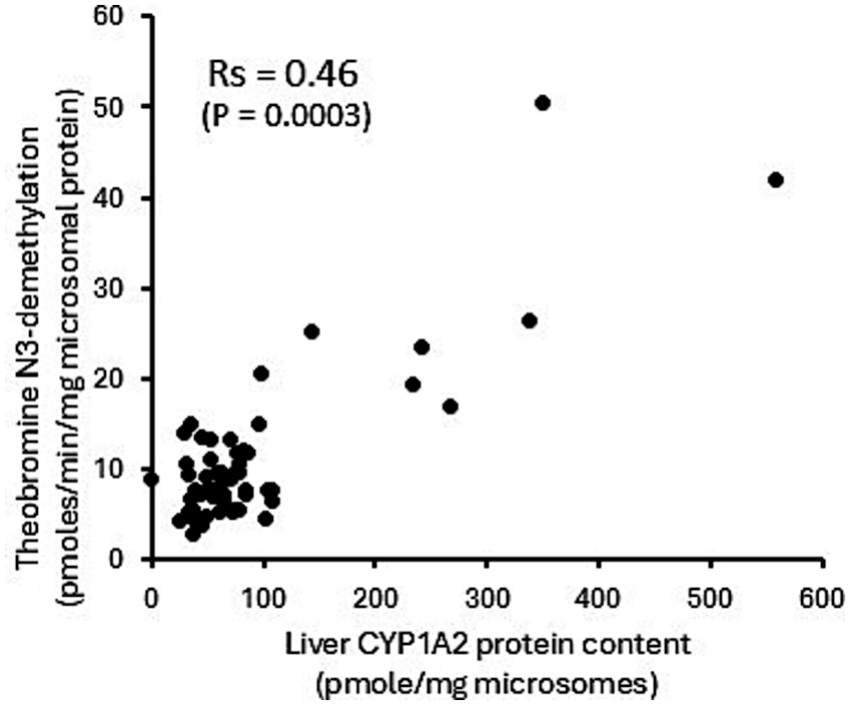
Theobromine N3-demethylation activities measured in individual dog liver bank microsome samples. There is moderate statistically significant correlation (*R*_s_ = 0.46; *p* = 0.0003) with CYP1A2 protein concentration.

The potential utility of theobromine N3-demethylation as a selective probe for CYP1A2 function in canine liver was evaluated using recombinant CYPs, liver microsomes from dogs treated with CYP selective inducers, and correlation of activities in the canine liver bank with CYP1A2 protein content. As shown in Figure 4A, only CYP1A1 (14 ± 2 pmoles/min/nmole P450) and CYP1A2 (25 ± 13 pmoles/min/nmole P450) showed theobromine N3-demethylation activity, while other CYPs showed no activity (lower limit of detection equivalent to 0.5 pmoles/min/nmole P450). Furthermore, theobromine N3-demethylation activity measured in liver microsomes from dogs treated with β-napthoflavone showed over 10-fold higher activity compared with microsomes from dogs treated with the vehicle (corn oil). None of the other inducing agents evaluated (rifampin, phenobarbital, or clofibric acid) showed any effect on theobromine N3-demethylation activities compared with control treatments (corn oil and saline) (Figure 4B). Finally, theobromine N3-demethylation activities measured in individual dog liver bank microsome samples showed a moderate statistically significant correlation (*R*_s_ = 0.46; *p* = 0.0003) with CYP1A2 protein concentration (Figure 5). Theobromine N3-demethylation activities and CYP1A2 protein content for individual liver samples are provided in Supplementary Table 1.

## 4 Discussion

The results of this study did not support the hypothesis that higher CYP1A2 copy number is associated with higher liver CYP1A2 protein content and enzyme activity. Interestingly, we did note that all six liver samples with the highest CYP1A2 protein content (Figure 2A) were also determined to have three CYP1A2 gene copies. Nevertheless, livers with more than three copies (four or five) showed lower rather than higher CYP1A2 protein content. These findings add to findings in a previous report (23) that found no association between CYP1A2 gene copy number and mRNA albeit using a smaller sample size (*N* = 18) than used here (*N* = 58). Consequently, the limited evidence so far suggests that CYP1A2 gene copy number may not be a sensitive indicator of rapid CYP1A2 metabolism in dogs.

Other genetic and non-genetic factors may explain interindividual variability in CYP1A2 metabolism. CYP1A2 expression is readily induced following exposure to aryl hydrocarbon environmental mediated induction (cigarette smoke) and certain drugs. Consequently the six livers noted above may have been obtained from dogs that had been exposed to these compounds in the diet or environment. It was also suggested by Wang et al. (23) that the effect of increased gene copy number may only become apparent following exposure to CYP1A2 inducers. This could be evaluated in the future by determining the extent of increase in CYP1A2 metabolism *in vivo* following administration of an inducer such as omeprazole.

Evidence supporting theobromine N-3 demethylation as an *in vitro* probe for canine CYP1A2 included demonstrating a lack of metabolic activity for all major constitutive drug metabolizing CYP isoforms other than CYP1A2 and observing substantial induction of enzyme activity by the aryl hydrocarbon inducer (polycyclic aromatic hydrocarbons: cigarette smoke) and β-napthoflavone. Although theobromine N-3 demethylation activities measured in the liver bank samples were correlated with CYP1A2 protein activity, the strength of correlation was relatively modest. Furthermore, enzyme activity was observed in the liver sample with the CYP1A2 p.373 X/X genotype (Figure 2B) despite that sample lacking any detectable CYP1A2 protein. Consequently, CYPs other than those we screened for activity must contribute to theobromine N-3 demethylation in dog liver. Several possibilities exist including canine CYP2E1, CYP2A13, and CYP2A25. Human CYP2E1 showed some theobromine N-3 demethylation activity in a previous study (22). Canine CYP2A13 and CYP2A25 are poorly studied CYPs that are expressed in dog liver (26). CYP2A13 can metabolize phenacetin and may contribute to the lower selectivity of phenacetin as a CYP1A2 probe in dogs compared with humans. Unfortunately, recombinant canine CYP2E1, CYP2A13, and CYP2A25 are not commercially available and were not evaluated in this study.

There are several limitations to this study that should be mentioned. The liver bank samples we used are relatively heterogeneous, being derived from various dog breeds. The differences not being significant can also be related to the small number of livers and possible bias in the sampling, and additionally how some of the breed differences seen can be related to geographic location of the populations (the Japanese beagles for example), their establishment and possible genetic drift. The distribution of the stop codon mutation across multiple breeds may be the result of its emergence early during, or even prior to, the formation of the breeds that have been established to date (approximately 350 breeds) (27). This could add additional variability that might obscure any CYP1A2 copy number variation. Although we attempted to control for this by performing additional subgroup analyses using livers derived from beagle dogs (*N* = 25), we were unable to demonstrate any correlation with genotype. An additional limitation is that we have no information regarding the exposure of the dogs to enzyme inducers (secondhand smoking), the diet or environment which might explain why six liver samples showed high CYP1A2 expression. Finally, a higher number of liver samples studied may have increased the ability to discern more subtle genotype effects. Specifically, we only were able to identify 6 dogs with the highest number of gene copies (5). Unfortunately, like other drug metabolizing enzymes, CYP1A2 activity, as has been determined in humans; is highly variable and other factors besides the genetic makeup (epigenetics, sex, disease) can play a role and since this enzyme has not been extensively studied in the dog, we are trying to elucidate differences and similarities seen in human pharmacogenetics. Further studies are necessary to determine a more direct impact of CYP1A2 function in veterinary clinical pharmacology. Determination of a more specific *in vitro* probe is fundamental to continue delving into the contribution, together with a more detailed understanding of the breed differences, geographical location, sex and thorough medical history (exposure to smoke, drugs given at the moment of testing) that can affect function of the enzyme.

## 5 Conclusion

CYP1A2 gene copy number is not a strong predictor of fast CYP1A2 metabolism in dog liver. Further work is needed to determine whether the duplicated CYP1A2 gene copies are functional and contribute to CYP1A2 mediated metabolism. Additional work is also needed to determine whether additional CYP isoforms contribute to theobromine N-3 demethylation in canine liver, thereby limiting the CYP1A2 selectivity of this activity.

## Data Availability

The original contributions presented in the study are included in the article/Supplementary material, further inquiries can be directed to the corresponding author.

## Supplementary material

SUPPLEMENTARY FIGURE 1 Sequence of the reference gene, UGT1A “gblock”.
