# Supplementary figures and images for: Evaluation of the relationship between cytochrome P450 (CYP) 1A2 gene copy number variation and CYP1A2 protein content and enzyme activity in canine liver
Source: Front Vet Sci. 2025 Jul 22;12:1511341. doi: 10.3389/fvets.2025.1511341 (PMC12322894; doi:10.3389/fvets.2025.1511341)

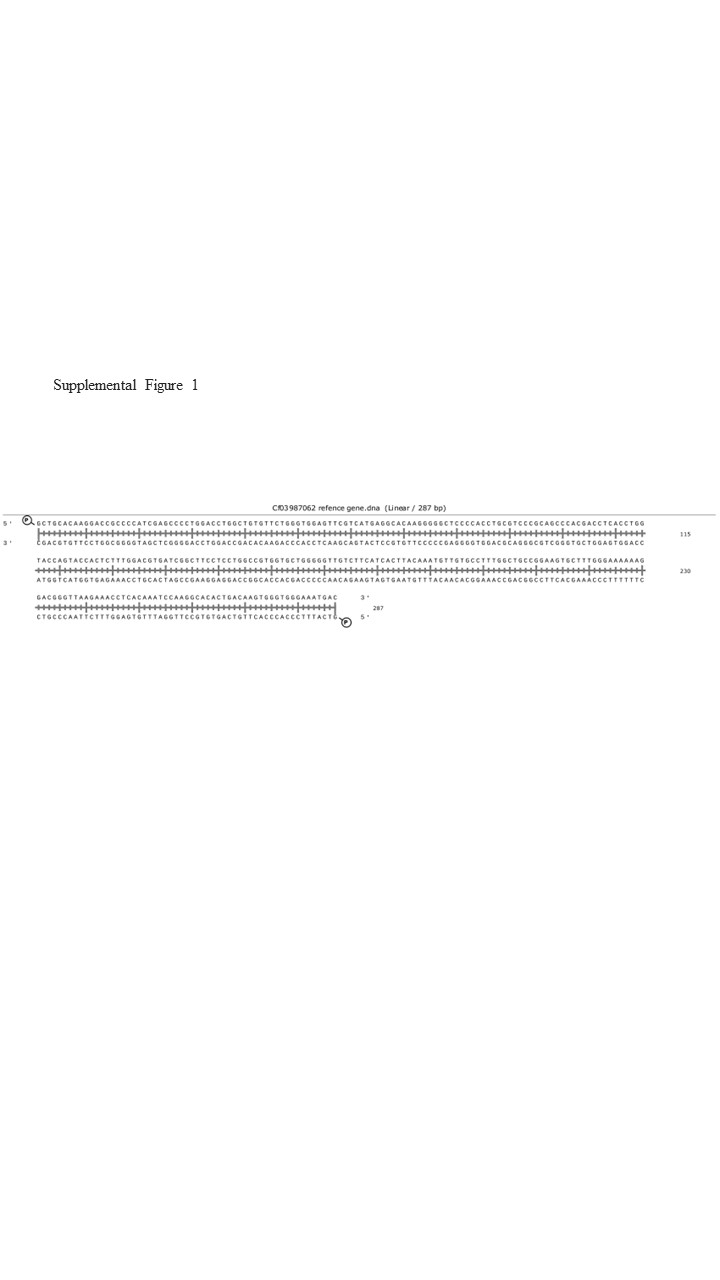

Supplement: SUPPLEMENTARY FIGURE 1 — Sequence of the reference gene, UGT1A “gblock”. [file Image_1.jpeg]
