# Supplementary material for: Evaluation of the relationship between cytochrome P450 (CYP) 1A2 gene copy number variation and CYP1A2 protein content and enzyme activity in canine liver
Source: Front Vet Sci. 2025 Jul 22;12:1511341. doi: 10.3389/fvets.2025.1511341 (PMC12322894; doi:10.3389/fvets.2025.1511341)
Supplement: Supplementary file 2 [file Table_1.docx]

Supplemental Table 1: Data for each individual dog liver bank samples (N=59)

| **ID  Sample** | **Mean  Delta CT** | **STDEV of Delta CT** | **Liver CYP1A2  protein content  (pmole/mg microsomal protein)** | **Liver CYP1A2 probe activity (Theobromine N-demethylation to 7-methylxanthine) (pmoles/min/mg microsomal protein)** | **CYP1A2stop  (R=Arginine; X=stop codon) Genotype** | **Estimated  copy  number** | **Breed** | **Sex** |
| --- | --- | --- | --- | --- | --- | --- | --- | --- |
| DL-01 | 0.53 | 0.23 | 78.7 | 5.5 | R/R | 4 | Research Beagle | M |
| DL-02 | -0.48 | 0.28 | 64.9 | 7.2 | R/X | 2 | Research Beagle | M |
| DL-03 | 0.53 | 0.19 | 73.8 | 5.5 | R/R | 4 | NGA Greyhound | M |
| DL-04 | 0.74 | 0.27 | 25.0 | 4.4 | R/X | 4 | NGA Greyhound | M |
| DL-05 | 0.22 | 0.20 | 266.5 | 17.1 | R/R | 3 | Mixed | F |
| DL-06 | 0.60 | 0.24 | 108.0 | 7.8 | R/R | 4 | Mixed | F |
| DL-07 | 0.54 | 0.28 | 102.8 | 4.6 | R/R | 4 | Mixed | F |
| DL-08 | -0.33 | 0.20 | 83.7 | 7.8 | R/R | 2 | NGA Greyhound | M |
| DL-09 | 1.72 | 0.47 | 48.4 | 4.9 | R/R | 5 | NGA Greyhound | F |
| DL-10 | 1.66 | 0.22 | 44.5 | 4.0 | R/R | 5 | NGA Greyhound | F |
| DL-11 |  |  | 61.5 | 5.3 | No DNA | - | Mixed | F |
| DL-12 | 0.27 | 0.26 | 37.8 | 5.7 | R/R | 4 | Mixed | M |
| DL-13 | 1.36 | 0.24 | 37.0 | 4.8 | R/R | 5 | Mixed | M |
| DL-14 | 0.50 | 0.24 | 35.5 | 5.6 | R/R | 4 | Mixed | F |
| DL-15 | 0.17 | 0.23 | 39.0 | 4.2 | R/X | 3 | Research Beagle | M |
| DL-16 | 0.11 | 0.24 | 49.9 | 7.9 | R/R | 3 | Research Beagle | M |
| DL-17 | 1.02 | 0.41 | 36.2 | 2.8 | R/R | 5 | Mixed | M |
| DL-18 | 0.64 | 0.30 | 71.1 | 9.0 | R/R | 4 | Mixed | M |
| DL-19 | 0.67 | 0.25 | 97.9 | 20.5 | R/R | 4 | Mixed | M |
| DL-20 | 0.11 | 0.26 | 107.1 | 6.5 | R/X | 3 | Research Beagle | M |
| DL-21 | 0.45 | 0.27 | 40.1 | 4.6 | R/R | 4 | Mixed | F |
| DL-22 | -0.40 | 0.25 | 73.3 | 9.5 | R/R | 2 | Mixed | M |
| DL-23 | 1.60 | 0.35 | 84.3 | 7.3 | R/R | 5 | Chihuahua | M |
| DL-24 | 0.12 | 0.22 | 44.9 | 13.7 | R/R | 3 | Chihuahua | M |
| DL-25 | 1.21 | 1.00 | 86.1 | 11.8 | R/R | 5 | Chihuahua | M |
| DL-26 | 0.50 | 0.20 | 35.6 | 6.6 | R/R | 4 | Chihuahua | F |
| DL-27 | 0.11 | 0.30 | 32.7 | 9.4 | R/X | 3 | Mixed | M |
| DL-28 | -0.06 | 0.21 | 234.1 | 19.4 | R/X | 3 | Research Beagle | F |
| DL-29 | 0.07 | 0.29 | 349.4 | 50.5 | R/R | 3 | Research Beagle | F |
| DL-30 | -0.05 | 0.28 | 337.8 | 26.4 | R/X | 3 | Research Beagle | M |
| DL-31 | 0.00 | 0.20 | 557.3 | 42.0 | R/R | 3 | Research Beagle | M |
| DL-32 | -0.54 | 0.21 | 143.6 | 25.2 | R/R | 2 | Research Beagle | M |
| DL-33 | 0.13 | 0.31 | 241.8 | 23.4 | R/X | 3 | Research Beagle | F |
| DL-34 | 0.65 | 0.21 | 43.7 | 7.4 | R/R | 4 | Research Hound | F |
| DL-35 | 0.43 | 0.22 | 76.0 | 11.8 | R/R | 4 | Research Hound | F |
| DL-36 | 0.68 | 0.20 | 63.6 | 6.6 | R/R | 4 | Research Hound | F |
| DL-37 | 0.11 | 0.19 | 40.0 | 7.6 | R/R | 3 | Research Hound | F |
| DL-38 | -0.02 | 0.21 | 31.6 | 10.5 | R/R | 3 | Research Hound | F |
| DL-39 | 0.00 | 0.21 | 52.7 | 11.2 | R/R | 3 | Research Hound | F |
| DL-40 | 0.06 | 0.19 | 33.4 | 5.3 | R/R | 3 | Research Hound | F |
| DL-41 | -0.05 | 0.22 | 104.0 | 7.8 | R/R | 3 | Research Hound | F |
| DL-42 | 0.42 | 0.25 | 42.9 | 7.2 | R/R | 4 | Research Hound | F |
| DL-43 | -0.30 | 0.12 | 53.9 | 7.1 | R/R | 2 | Research Hound | F |
| DL-44 | 0.46 | 0.25 | 58.3 | 8.0 | R/R | 4 | Research Hound | F |
| DL-45 | 0.03 | 0.24 | 72.9 | 5.4 | R/R | 3 | Research Hound | F |
| DL-46 | 0.50 | 0.21 | 63.2 | 9.7 | R/R | 4 | Research Beagle | M |
| DL-47 | 0.41 | 0.38 | 72.5 | 9.3 | R/R | 4 | Research Beagle | M |
| DL-48 | 0.49 | 0.26 | 63.9 | 8.7 | R/R | 4 | Research Beagle | M |
| DL-49 | 0.52 | 0.23 | 48.1 | 9.1 | R/R | 4 | Research Beagle | M |
| DL-50 | 0.32 | 0.12 | 57.7 | 9.7 | R/R | 4 | Research Beagle | M |
| DL-51 | 0.05 | 0.23 | 53.5 | 13.3 | R/X | 3 | Research Beagle | M |
| DL-52 | -0.42 | 0.20 | 96.6 | 15.0 | R/R | 2 | Research Beagle | M |
| DL-53 | -0.36 | 0.22 | 30.1 | 14.2 | R/X | 2 | Research Beagle | M |
| DL-54 | 0.21 | 0.19 | 35.6 | 15.0 | R/R | 3 | Research Beagle | M |
| DL-55 | -0.47 | 0.22 | 0.0 | 9.1 | X/X | 2 | Research Beagle | M |
| DL-56 | 0.28 | 0.23 | 82.0 | 12.0 | R/R | 4 | Research Beagle | M |
| DL-57 | 0.18 | 0.29 | 77.4 | 9.6 | R/R | 3 | Research Beagle | F |
| DL-58 | -0.17 | 0.23 | 77.7 | 10.6 | R/X | 2 | Research Beagle | F |
| DL-59 | 0.27 | 0.33 | 70.9 | 13.2 | R/R | 4 | Research Beagle | F |

Supplemental Table 2: CYP1A2 microsomal protein content measured in dog liver bank samples lacking the CYP1A2 p.373 stop codon mutation (N = 46) with 2, 3, 4, or 5 CYP1A2 gene copies. Shown are the median, 25^th^ to 75^th^ percentile microsomal CYP1A2 protein content values and the P value for comparison between copy number groups by ANOVA on Ranks. There is not a statistically significant difference between copy number groups (P > 0.05).

| **Estimated CYP1A2**  **Gene Copy Number** | **Microsomal CYP1A2 protein content**  **(pmole/ mg microsomal protein)** | | | | |
| --- | --- | --- | --- | --- | --- |
|  | **N** | **Median** | **25%** | **75%** | **P value** |
| **2** | 5 | 84 | 64 | 120 | 0.38 |
| **3** | 13 | 53 | 38 | 185 |  |
| **4** | 22 | 64 | 43 | 77 |  |
| **5** | 6 | 46 | 37 | 85 |  |

Supplemental Table 3: Microsomal theobromine N3-demethylation activities measured in dog liver bank samples lacking the CYP1A2 p.373 stop codon mutation (N = 46) with 2, 3, 4, or 5 CYP1A2 gene copies. Shown are the median, 25^th^ to 75^th^ percentile theobromine N3-demethylation activity values and the P value for comparison between copy number groups by ANOVA on Ranks. There is a statistically significant difference between copy number groups (P < 0.05). Pairwise group comparisons using Dunn’s test did not identify groups that were significantly different (P > 0.05).

| **Estimated CYP1A2**  **Gene Copy Number** | **Theobromine N3-demethylation activity**  **(pmole / min / mg microsomal protein)** | | | | |
| --- | --- | --- | --- | --- | --- |
|  | **N** | **Median** | **25%** | **75%** | **P value** |
| **2** | 5 | 9.5 | 7.4 | 20.1 | 0.023 |
| **3** | 13 | 10.5 | 7.7 | 16.0 |  |
| **4** | 22 | 7.9 | 5.6 | 9.7 |  |
| **5** | 6 | 4.8 | 3.7 | 8.4 |  |

Supplemental Table 4: CYP1A2 microsomal protein content measured in dog liver bank samples from beagle dogs lacking the CYP1A2 p.373 stop codon variant (N = 15) with 2, 3, 4, or 5 CYP1A2 gene copies. Shown are the median, 25^th^ to 75^th^ percentile microsomal CYP1A2 protein content values and the P value for comparison between copy number groups by ANOVA on Ranks. There is not a statistically significant difference between copy number groups (P > 0.05).

| **Estimated CYP1A2**  **Gene Copy Number** | **Microsomal CYP1A2 protein content**  **(pmole/ mg microsomal protein)** | | | | |
| --- | --- | --- | --- | --- | --- |
|  | **N** | **Median** | **25%** | **75%** | **P value** |
| **2** | 2 | 120 | 97 | 144 | 0.24 |
| **3** | 5 | 77 | 43 | 453 |  |
| **4** | 8 | 67 | 59 | 77 |  |

Supplemental Table 5: Microsomal theobromine N3-demethylation activities measured in dog liver bank samples from beagle dogs lacking the CYP1A2 p.373 stop codon variant (N = 15) with 2, 3, 4, or 5 CYP1A2 gene copies. Shown are the median, 25^th^ to 75^th^ percentile theobromine N3-demethylation activity values and the P value for comparison between copy number groups by ANOVA on Ranks. There is not a statistically significant difference between copy number groups (P > 0.05).

| **Estimated CYP1A2**  **Gene Copy Number** | **Theobromine N3-demethylation activity**  **(pmole / min / mg microsomal protein)** | | | | |
| --- | --- | --- | --- | --- | --- |
|  | **N** | **Median** | **25%** | **75%** | **P value** |
| **2** | 2 | 20.1 | 15.0 | 25.2 | 0.11 |
| **3** | 5 | 15.0 | 8.7 | 46.3 |  |
| **4** | 8 | 9.5 | 8.8 | 11.4 |  |
